# Dynamic control of *ERG20* expression combined with minimized endogenous downstream metabolism contributes to the improvement of geraniol production in *Saccharomyces cerevisiae*

**DOI:** 10.1186/s12934-017-0641-9

**Published:** 2017-01-31

**Authors:** Jianzhi Zhao, Chen Li, Yan Zhang, Yu Shen, Jin Hou, Xiaoming Bao

**Affiliations:** 1 State Key Laboratory of Microbial Technology, School of Life Science, Shandong University, Jinan, 250100 China; 2 Shandong Provincial Key Laboratory of Microbial Engineering, School of Bioengineering, QiLu University of Technology, Jinan, 250353 China

**Keywords:** Monoterpene, Geraniol, Geranyl diphosphate, *ERG20*, *Saccharomyces cerevisiae*

## Abstract

**Background:**

Microbial production of monoterpenes provides a promising substitute for traditional chemical-based methods, but their production is lagging compared with sesquiterpenes. Geraniol, a valuable monoterpene alcohol, is widely used in cosmetic, perfume, pharmaceutical and it is also a potential gasoline alternative. Previously, we constructed a geraniol production strain by engineering the mevalonate pathway together with the expression of a high-activity geraniol synthase.

**Results:**

In this study, we further improved the geraniol production through reducing the endogenous metabolism of geraniol and controlling the precursor geranyl diphosphate flux distribution. The deletion of *OYE2* (encoding an NADPH oxidoreductase) or *ATF1* (encoding an alcohol acetyltransferase) both involving endogenous conversion of geraniol to other terpenoids, improved geraniol production by 1.7-fold or 1.6-fold in batch fermentation, respectively. In addition, we found that direct down-regulation of *ERG20* expression, the branch point regulating geranyl diphosphate flux, does not improve geraniol production. Therefore, we explored dynamic control of *ERG20* expression to redistribute the precursor geranyl diphosphate flux and achieved a 3.4-fold increase in geraniol production after optimizing carbon source feeding. Furthermore, the combination of dynamic control of *ERG20* expression and *OYE2* deletion in *LEU2* prototrophic strain increased geraniol production up to 1.69 g/L with pure ethanol feeding in fed-batch fermentation, which is the highest reported production in engineered yeast.

**Conclusion:**

An efficient geraniol production platform was established by reducing the endogenous metabolism of geraniol and by controlling the flux distribution of the precursor geranyl diphosphate. The present work also provides a production basis to synthesis geraniol-derived chemicals, such as monoterpene indole alkaloids.

**Electronic supplementary material:**

The online version of this article (doi:10.1186/s12934-017-0641-9) contains supplementary material, which is available to authorized users.

## Background

In nature, isoprenoids are a large group of diverse compounds, that are biosynthesized via both the cytosolic mevalonate (MVA) pathway and the plastidial 2C-methyl-D-erythrtiol 4-phosphate (MEP) pathway in plants and most bacteria, or the mevalonate (MVA) pathway in animals and eukaryotes [1]. The universal precursors of isoprenoids, isopentenyl diphosphate (IPP) and dimethylallyl pyrophosphate (DMAPP), are derived from both pathways. Among them, monoterpenes are a particularly interesting subset of this family, which has been widely used as flavors, fragrances and pharmaceuticals as well as potential fuels [2, 3]. Traditionally, monoterpenes and their derivatives are produced from plants in low amounts, and the extraction process from plants is costly and highly dependent on the availability of raw materials. Engineering microbial organisms for monoterpene synthesis provides a potential effective route for their production.

**Fig. 1:**
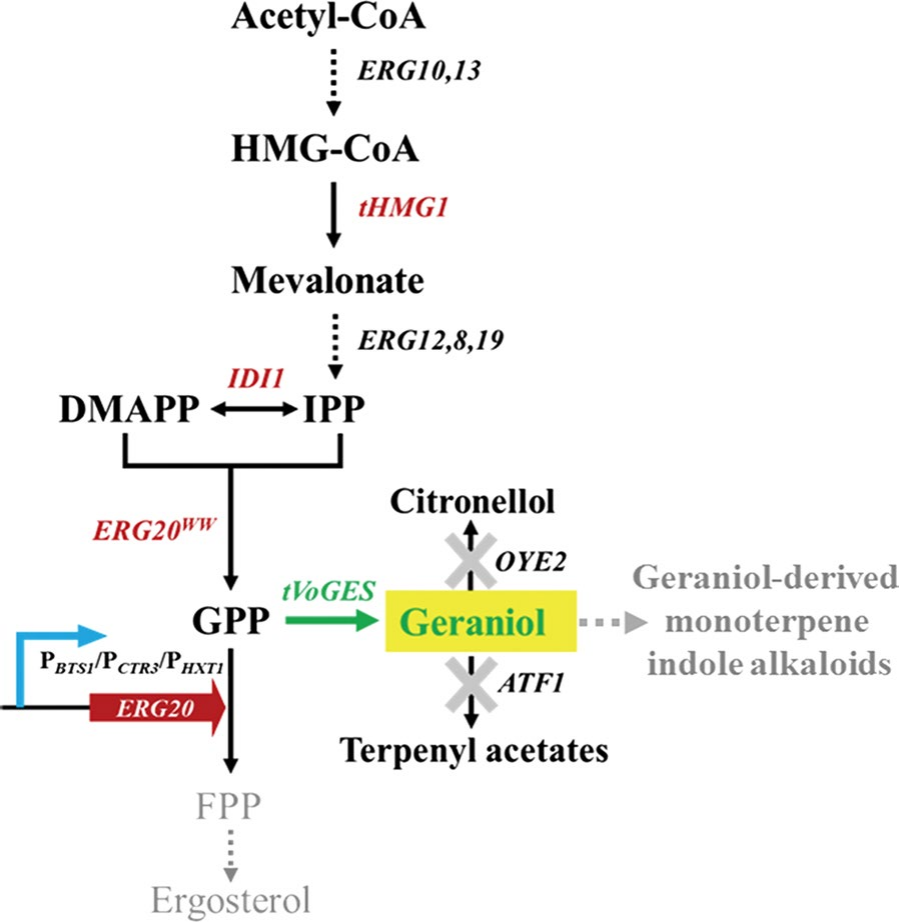
Schematic overview of geraniol biosynthesis based on the mevalonate (MVA) pathway in *S. cerevisiae*. The endogenous genes *tHMG1*, *IDI1* and *ERG20* ^*WW*^ and heterologous gene tVoGES encoding a truncated version of geraniol synthase from *Valeriana officinalis* were overexpressed to improve geraniol production in our previous work. The native promoter of *ERG20* was replaced with the *CTR3* promoter, *BTS1* promoter or *HXT1* promoter. Other engineered genes in this pathway: *OYE2*, NADPH oxidoreductase and *ATF1*, alcohol acetyltransferase. *HMG-CoA* 3-hydroxy-3-methylglutaryl coenzyme A, *IPP* isopentenyl pyrophosphate, *DMAPP* dimethylallyl pyrophosphate, *GPP* geranyl diphosphate, *FPP* farnesyl diphosphate

Monoterpene geraniol (*trans*-3,7-dimethyl-2,6-octadien-1-ol; C_10_H_18_O), an acyclic monoterpene, is typically generated from aromatic plants and has many applications in perfume, pharmaceutical, and other chemical industries [4]. Geraniol is also considered as a promising biofuel due to its high energy content, low hygroscopicity and low volatility in comparison with ethanol [5]. In addition, a recent study reported that geraniol can undergo an 11-step heterologous biosynthetic pathway to form strictosidine, which is the common intermediate for production of the anticancer agent monoterpene indole alkaloids (MIAs) [6]. Given its low yield in aromatic plants, geraniol has been successfully synthesized in *Escherichia coli* and *Saccharomyces cerevisiae* through metabolic engineering strategies [7–11]. *S. cerevisiae* has been used widely as a cell factory to produce a diversity of terpenes, and many of them have achieved significant yields [12–15]. However, monoterpene production has been achieved only at low levels thus far [8, 16, 17]. There are two challenges for high level monoterpenes production. One is the availability of intracellular geranyl diphosphate (GPP) precursor, and the other is the toxicity of monoterpenes to cells [18, 19]. Most of monoterpenes are generated from the precursor GPP which is formed by condensation of one molecule of IPP with one molecule of its isomer DMAPP (Fig. 1). Unlike plants, *S. cerevisiae* does not supply enough GPP for production due to the lack of a specific GPP synthase (GPPS). In *S. cerevisiae*, the *ERG20* gene of the endogenous MVA pathway encodes a farnesyl pyrophosphate synthase (FPPS) that, despite having both GPP and FPP synthase activity, only releases a very low amount of GPP from its catalytic site [20]. To increase the flux to GPP, a set of *ERG20* mutants was constructed to screen for GPPS preference mutants. *ERG20* ^*K197G*^, the most effective mutant, improved geraniol production to 5 mg/L while expressing geraniol synthase (GES) from *Ocimum basilicum* in *S. cerevisiae* [8]. Liu et al. obtained 36 mg/L geraniol by overexpressing two key rate-limiting genes, *tHMG1* encoding a truncated version of 3-hydroxy-3-methylglutaryl coenzyme A and *IDI1* encoding IPP isomerase, to enhance MVA pathway flux and by overexpressing *MAF1*, a negative regulator of *MOD5* encoding tRNA isopentenyltransferase, to decrease the flux to tRNA biosynthesis [10]. In addition, Ignea et al. demonstrated that the double mutant Erg20p (F96W-N127W) had a strong dominant negative ability to decrease the FPPS function of Erg20p, increasing production of the monoterpene sabinene by 10.4-fold to 0.53 mg/L in an industrial diploid strain [16]. In our previous work, we further improved geraniol production through enhancing MVA pathway flux, screening different sources of GESs and GPPSs, and designing fusion proteins of GES and GPPS. Geraniol of 293 mg/L production was achieved in fed-batch cultivation, which is the highest monoterpene production in *S. cerevisiae* ever reported [21]. However, it is still lower than the production of many other terpenes in *S. cerevisiae* [12, 22, 23].

In addition to the low GPP pool, the toxicity to microorganisms of monoterpenes also largely limits their microbial production [2, 24]. To alleviate the toxicity of monoterpenes, a two-phase fermentation system has been extensively applied by adding a non-toxic extractive solvent (e.g., dodecane) [2]. In addition, monoterpenols, usually undergo biotransformation to other terpenoids in aromatic plants, as well as in some wine yeasts [25, 26]. A recent study demonstrated that geraniol is converted into citronellol under the catalysis of the enzyme *OYE2*, and it is acetylated by *ATF1* encoding alcohol acetyltransferase during wine fermentation by *S. cerevisiae* [27]. Intracellular bioconversion of geraniol may be a self-defense response to avoid the toxicity of monoterpenes. In addition, introduction of a heterologous biosynthetic pathway and the overexpression of an endogenous pathway often lead to the accumulation of some intermediate metabolites, that may interfere with the native regulation of metabolic flux and increase metabolic burden, resulting in overproduction of some biosynthetic enzymes and accumulation of some toxic intermediate metabolites. Therefore, a dynamic control system that can sense environmental changes such as metabolic intermediates or nutrient concentrations can be used to avoid the toxicity of intermediate metabolites [28–30].

Previously, we achieved a significant increase in geraniol production through expressing geraniol synthase from *Valeriana officinalis* and regulating GPP synthesis [21]. In this study, we further engineered strains through dynamic control of *ERG20* to fine-tune the GPP flux combined with minimized geraniol endogenous conversion. Combining these strategies together with *LEU2* auxotrophic complementation, the final geraniol production reached 1.69 g/L in fed-batch fermentation, which is the highest reported production in yeast.

## Results

### Improving geraniol production by minimizing endogenous bioconversion

**Fig. 2:**
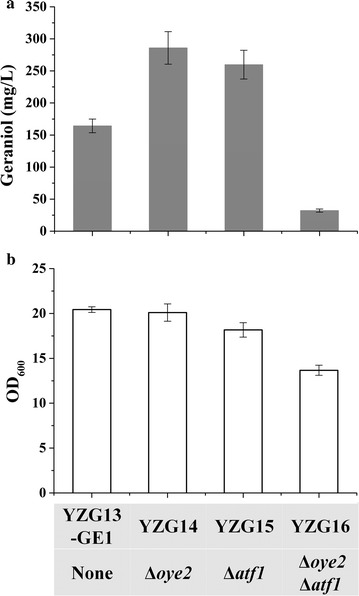
Production of geraniol by reducing endogenous conversion of geraniol in batch fermentation. **a** Production of geraniol in engineered strains. **b** Cell growth of engineered strains. The data shown are representative of duplicate experiments, and the *error bars* represent the standard deviation

Geraniol is the main precursor of monoterpenoids in aromatic plants [4]. It also has many derivatives including nerol, neral, geranylgeraniol, geranial, citronellol and terpenyl acetates [31]. Previous studies have demonstrated that wine yeasts are able to convert geraniol into other monoterpenoids, which influence the sensory properties of wine [26, 32]. During *S. cerevisiae* fermentation, Oye2p is the main enzyme involved in the conversion of geraniol to citronellol, and Atf1p is the main contributor to synthesis of terpenyl acetates from geraniol [27]. In our early study, an efficient *S. cerevisiae* strain YZG13-GE1 was constructed to produce geraniol from glucose through engineering of geraniol synthesis and optimizing GPP synthesis. To further improve geraniol production, we attempted to minimize endogenous metabolism of geraniol in *S. cerevisiae*. Thus, we deleted *OYE2* or *ATF1* in YZG13-GE1 to create YZG14 or YZG15, respectively. Batch fermentation was carried out in a two-phase fermentation system using dodecane as extractive solvent. Geraniol was produced at 285.9 mg/L by YZG14 with *OYE2* deletion, which was improved by 1.7-fold compared with the control strain (YZG13-GE1), and 259.8 mg/L was produced by YZG15 with *ATF1* deletion, which was improved by 1.6-fold compared with the control strain (YZG13-GE1) (Fig. 2a). The conversion of geraniol to citronellol was decreased by 55% in YZG14 (Δ*oye2*) (Additional file 1: Figure S1). In addition, we further investigated the effect of double deletion of *OYE2* and *ATF1* in YZG13-GE1 on geraniol production. The results showed that geraniol production dramatically decreased to 32.2 mg/L in YZG16 with *OYE2*-*ATF1* deletions (Fig. 2a). Cell growth was not affected in either single gene deletion strain (Δ*oye2* or Δ*atf1*) (Additional file 1: Figure S2), and geraniol yields were 57.8 mg/g DCW and 58.2 mg/g DCW, a 1.8-fold and 1.7-fold increase, respectively (Fig. 2b; Table 1). However, double deletion of *OYE2* and *ATF1* decreased the final biomass by 35%, and geraniol yield significantly dropped to 9.6 mg/g DCW (Fig. 2; Table 1).

**Table 1:** Geraniol yield of the engineered strains in batch fermentation

Strains^a^	Dry cell weight (g/L)	Geraniol yield (mg/g DCW)
YZG13-GE1	5.02 ± 0.31	32.69 ± 2.01
YZG14 (Δ*oye2*)	4.94 ± 0.28	57.83 ± 3.24
YZG15 (Δ*atf1*)	4.47 ± 0.22	58.16 ± 2.59
YZG16 (Δ*oye2*Δ*atf1*)	3.36 ± 0.34	9.58 ± 0.96
YZG17 (P_*BTS1*_-*ERG20*)	1.96 ± 0.10	20.58 ± 0.91
YZG18 (P_*CTR3*_-*ERG20*)	2.94 ± 0.23	16.61 ± 1.21
YZG19 (P_*HXT1*_-*ERG20*)	3.92 ± 0.26	37.87 ± 2.55

^a^Duplicate experiments were performed for each strain, and the error bars the represented the standard deviation

**Fig. 3:**
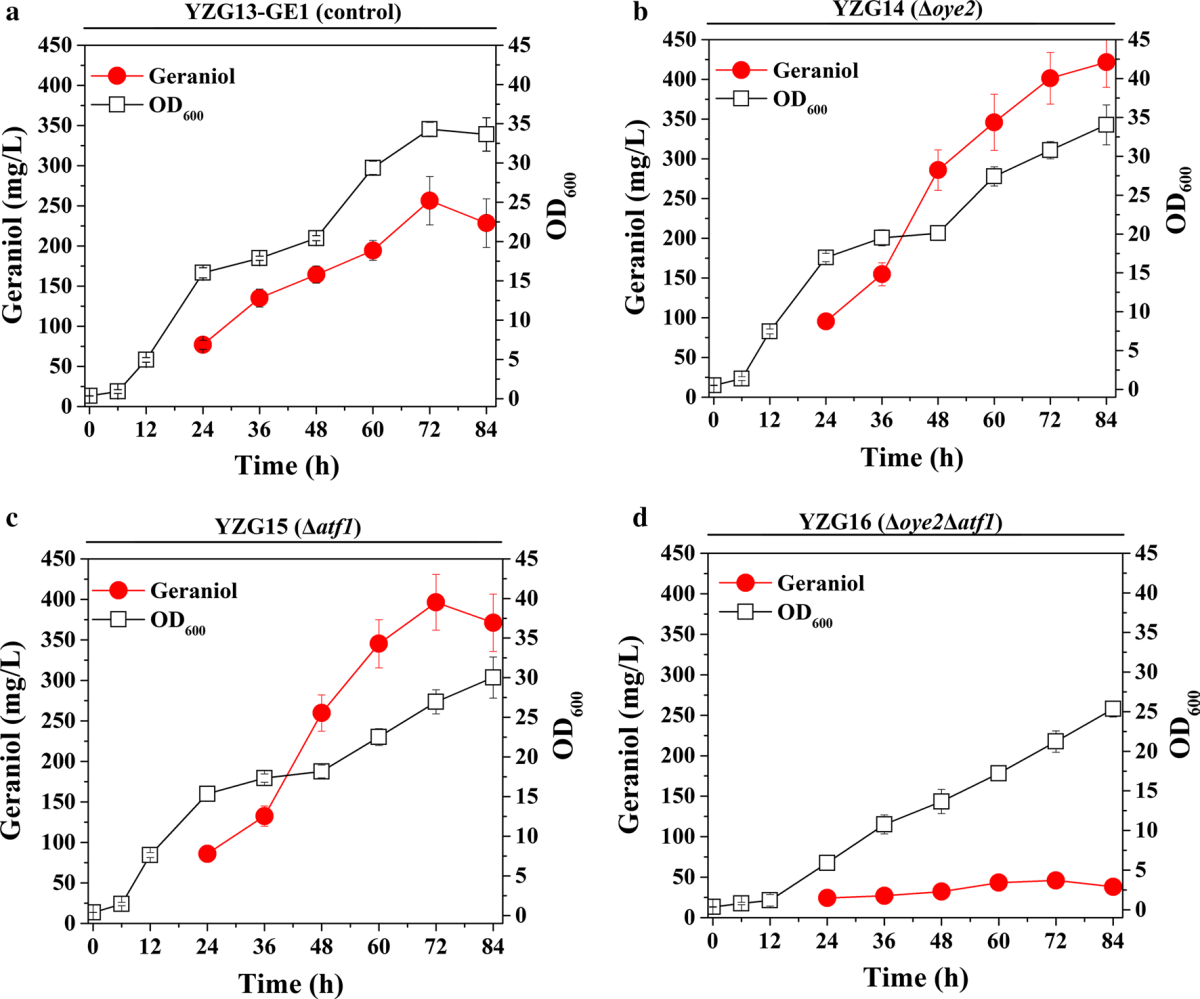
Fed-batch fermentation of YZG13-GE1 (control) (**a**), YZG14 (Δ*oye2*) (**b**), YZG15 (Δ*atf1*) (**c**) and YZG16 (Δ*oye2*Δ*atf1*) (**d**). Aerobic fed-batch fermentations were carried out by feeding 400 g/L glucose at 0.1/h feed rate. The data shown are representative of duplicate experiments, and the *error bars* represent the standard deviation

We also compared geraniol production in YZG14 (Δ*oye2*), YZG15 (Δ*atf1*) and YZG16 (Δ*oye2*Δ*atf1*) in fed-batch cultivation using glucose as sole carbon source. The cell growth of YZG14 (Δ*oye2*) and YZG15 (Δ*atf1*) exhibited a similar growth profile compared with the reference strain YZG13-GE1. YZG14 (Δ*oye2*) and YZG15 (Δ*atf1*) produced 421.7 and 371.1 mg/L of geraniol, which represented approximately 2.5-fold and 2.2-fold increases relative to YZG13-GE1 (256.4 mg/L), respectively (Fig. 3). However, geraniol production was dramatically decreased in double deletion of *OYE2* and *ATF1* strain, only producing 46.1 mg/L geraniol (Fig. 3d).

### The effect of *ERG20* expression control on geraniol production

**Fig. 4:**
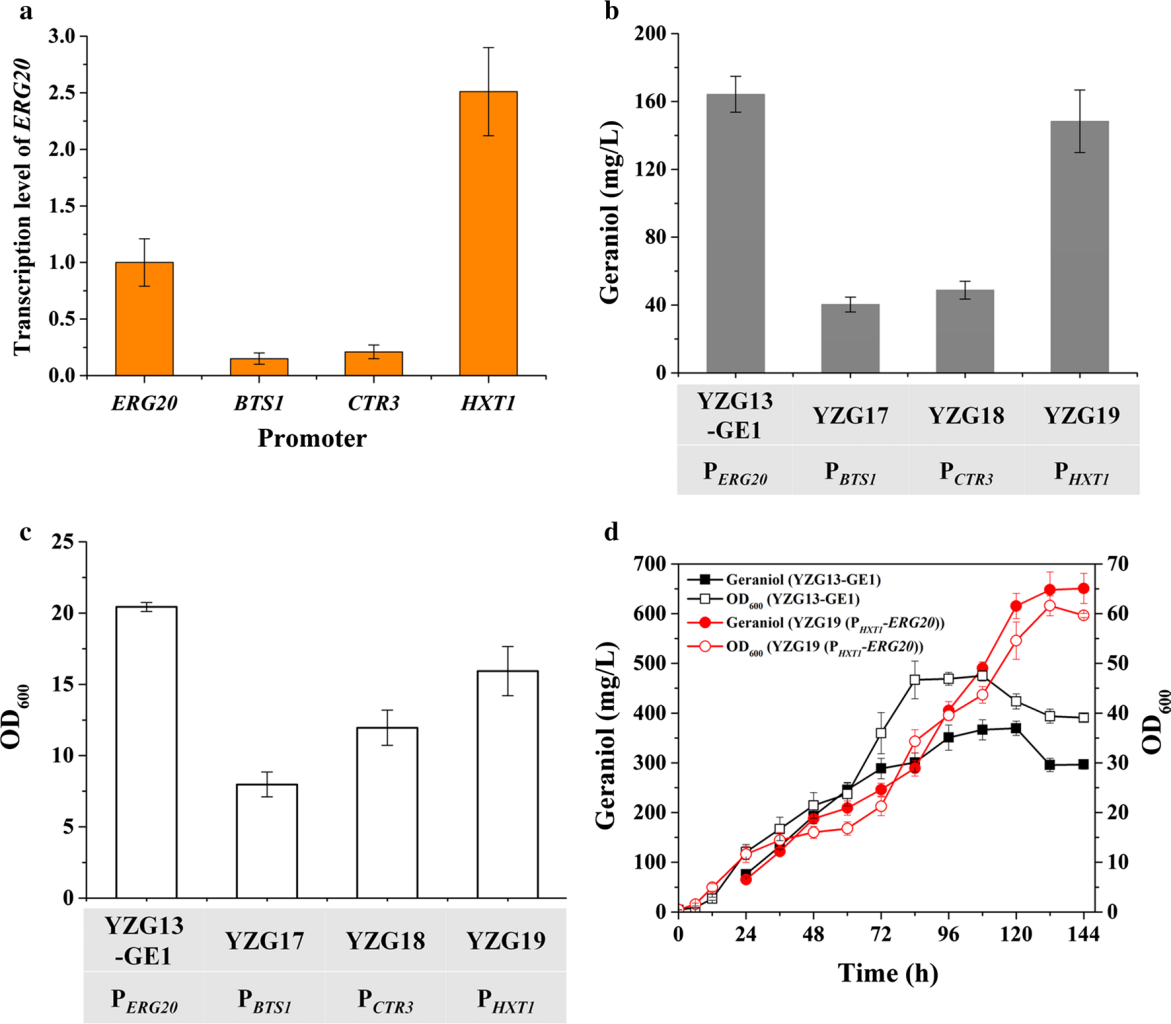
Production of geraniol in *ERG20* promoter replacement strains. **a** Transcription level of *ERG20* controlled by different promoters. **b** Production of geraniol in engineered strains in batch fermentation. **c** Cell growth of engineered strains in batch fermentation. **d** Geraniol production in YZG13-GE1 and *HXT1* promoter replacement strain (YZG19) in fed-batch fermentation. All strains were grown in SD medium with 2% glucose, and the cells were collected when OD_600_ reached 0.6 to extract mRNA for *ERG20* mRNA and transcription determination. Aerobic fed-batch fermentations were carried out by feeding a 50 g/L glucose and 350 g/L ethanol (1:7) mixture at 0.1/h feed rate. The data shown are representative of duplicate experiments, and the *error bars* represent the standard deviation

In *S. cerevisiae*, synthesis of both the monoterpene precursor GPP and sesquiterpene precursor FPP is catalyzed by a single enzyme, Erg20p, with FPP synthase preference. In the absence of a specific GPP synthase, many efforts have been made to increase the intercellular GPP pool. Expression of heterologous GPP synthase does not increase the GPP-derived products significantly [16, 21]. Although engineering Erg20p into a GPP synthase increased sabinene and geraniol production by 10.4-fold and 2.8-fold, respectively, the titer is still much lower than that of FPP-derived sesquiterpenes, demonstrating the importance of down-regulating FPP synthesis [10, 16]. In this study, the native *ERG20* promoter was first replaced with either the weak *BTS1* promoter or the copper repressible *CTR3* promoter. YZG13-GE1 containing the native *ERG20* promoter was used as a reference strain. The transcription level of *ERG20* using the *BTS1* promoter or *CTR3* promoter was reduced by 90 and 75% (Fig. 4a), respectively, in SD-URA-HIS medium with 2% glucose. Unexpectedly, the replacement of the *ERG20* promoter with that of *BTS1* or *CTR3* decreased geraniol production by 75.6 and 70.3%, resulting in 40.37 and 48.85 mg/L, respectively (Fig. 4b). The *BTS1* and *CTR3* promoters also decreased the final biomass by 60 and 40% (Fig. 4c), repectively. Previously, we also found that overexpression of *ERG20* reduced geraniol production [21]. These results demonstrated that both down-regulation and up-regulation of *ERG20* have a negative impact on geraniol production. The dynamic control of *ERG20* was then attempted by replacing the *ERG20* promoter with the glucose-sensing *HXT1* promoter. This promoter strength is higher than that of *ERG20* promoter when glucose is present in the medium (Fig. 4a), but it is repressed when the glucose concentration is low or absent (Additional file 1: Figure S3). We found that the geraniol titer and final biomass were not markedly affected by *HXT1* promoter replacement in batch cultivation, and the yield of geraniol increased slightly (37.9 mg/g DCW vs. 32.7 mg/g DCW) (Table 1). Considering that the *HXT1* promoter provides both high-glucose induction and low-glucose repression, we attempted both glucose and glucose/ethanol (1:7) mixture feeding strategies to control *ERG20* expression in fed-batch fermentation. Compared with the control strain, *HXT1* promoter replacement did not increase geraniol production when pure glucose was fed; geraniol production only increased slightly (data not shown). In contrast, when a glucose/ethanol (1:7) mixture was fed, the geraniol production achieved a 176% increase in the *HXT1* promoter replacement strain compared with the control strain, leading to 650.8 mg/L (Fig. 4d).

**Fig. 5:**
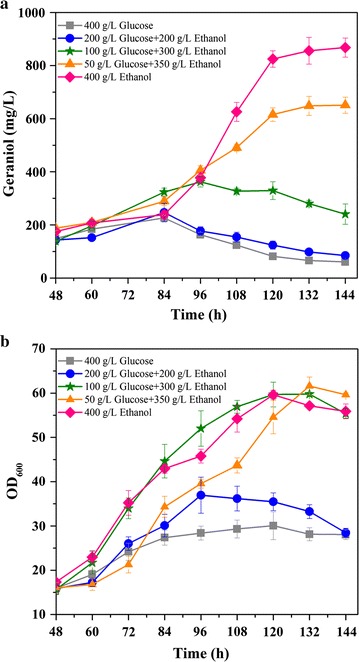
The effect of different carbon sources on geraniol production by YZG19 (P_*HXT1*_-*ERG20*) in fed-batch fermentation. **a** Production of geraniol by YZG19. **b** Cell growth of YZG19. Aerobic fed-batch fermentations were carried out by feeding different ratios of glucose and ethanol at 0.1/h feed rate. The data shown are representative of duplicate experiments, and the *error bars* represent the standard deviation

Previously, ethanol has been used as carbon for the production of hydrocortisone, amorpha-4,11-diene and resveratrol [33–35]. In this study, we further compared the impact of feeding different ratios of glucose and ethanol on geraniol production using YZG19 (P_*HXT1*_-*ERG20*). As shown in Fig. 5b, the cell growth of YZG19 (P_*HXT1*_-*ERG20*) gradually improved as the ethanol percentage in glucose/ethanol mixture increased, and the final production of geraniol also gradually increased. The highest production of geraniol was obtained when pure ethanol was fed, producing 867.7 mg/L of geraniol (Fig. 5a). Based on the results described above, the dynamic control of *ERG20* expression under the *HXT1* promoter together with optimizing the glucose and ethanol ratio improved the production of geraniol by 3.8-fold.

### Combining the dynamic control of *ERG20* and reduction of endogenous conversion together with *LEU2* complementation enabled a further increase in geraniol production

**Fig. 6:**
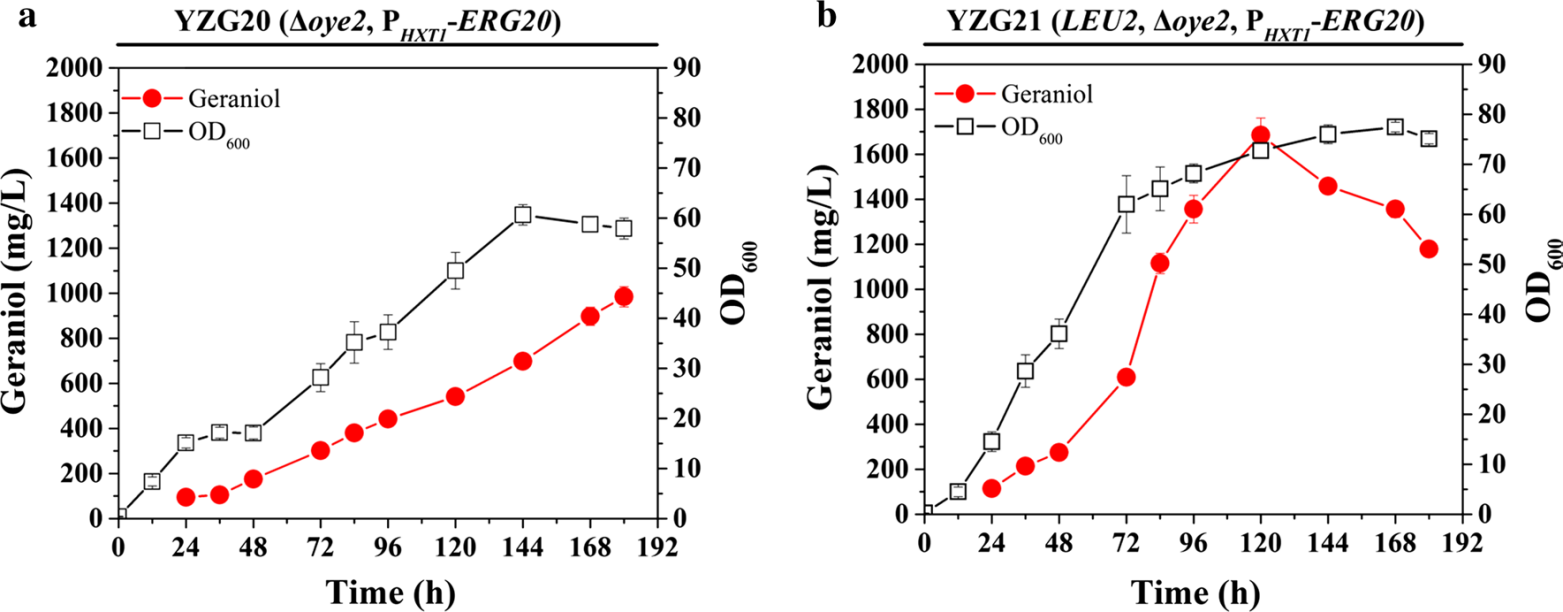
Fed-batch fermentation of YZG20 (Δ*oye2*, P_*HXT1*_-*ERG20*) (**a**) and YZG21 (*LEU2*, Δ*oye2*, P_*HXT1*_-*ERG20*) (**b**) using pure ethanol feeding. Aerobic fed-batch fermentations were carried out by feeding 400 g/L ethanol with 0.1/h feed rate. The data shown are representative of duplicate experiments, and the *error bars* represent standard deviations

To further improve geraniol production, we deleted the *OYE2* gene in YZG19 (P_*HXT1*_-*ERG20*) to generate YZG20 (Δ*oye2*, P_*HXT1*_-*ERG20*), which produced 984.6 mg/L of geraniol in fed-batch fermentation by feeding ethanol, a 14% increase over YZG19 (P_*HXT1*_-*ERG20*) (Fig. 6a). A recent study showed that leucine metabolism may be networked with isoprenoid biosynthesis, and leucine prototrophy was found to improve diterpenoid miltiradiene production [36]. We further engineered a strain with complemented *LEU2* auxotrophy, and final geraniol production was enhanced up to 1.69 g/L, a further 71% increase over YZG20 (Δ*oye2*, P_*HXT1*_-*ERG20*). Meanwhile, we noticed that *LEU2* complementation also improved cell growth and geraniol yield (Fig. 6b). The final geraniol production in this study was improved by 5.8-fold compared with our previous study.

## Discussion

Engineering *S. cerevisiae* as a cell factory is a promising and attractive route for rapid and inexpensive biosynthesis of terpenoids [12, 13, 22, 23, 34]. Over the last few years, most work has focused on the production of sesquiterpenes in microorganisms including *S. cerevisiae*. For instance, production of the sesquiterpene amorpha-4,11-diene, the precursor for the antimalarial agent artemisinin, has reached 40 g/L using engineered *S. cerevisiae* in fed-batch fermentation [12]. Both monoterpenes and sesquiterpenes are derived from the MVA pathway. However, monoterpene production in *S. cerevisiae* is much lower than that of sesquiterpenes, mainly due to the poor GPP precursor supply and the toxicity of monoterpenes in yeast. In this work, we further engineered production of the monoterpene geraniol through reduction of endogenous geraniol conversion, dynamic control of *ERG20* expression and leucine biosynthesis complementation based on YZG13-GE1, a geraniol-producing strain from our previous work [21]. Final geraniol production of 1.69 g/L was obtained, which to our knowledge is the highest level reported for engineered yeast.

Monoterpenes are highly toxic to many microorganisms. Cells may reduce their toxicity through the conversion of monoterpenes to other lower toxicity compounds by various specific or non-specific enzymes. In our previous study, we confirmed that 180 mg/L of geraniol in culture could strongly inhibit yeast cell growth [21]. Although two-phase extractive fermentation could effectively alleviate monoterpene toxicity in situ, the conversion of monoterpenes to other terpenoids is not avoided completely. In *S. cerevisiae*, a mechanism was proposed in which geraniol was first oxidized to geranial by *OYE2* encoding an NADPH oxidoreductase and its homologous gene *OYE3*, belonging to the old yellow enzyme family, and then was reduced to citronellol [25, 27, 37]. However, the oxido-reductases involved in the reaction of geranial to citronellol are still unknown. Of the two old yellow enzymes, *OYE2* was the main contributor to the reduction reaction. Except for citronellol, terpenyl acetates including citronellyl acetate and geranyl acetate are another group of geraniol-derived metabolites catalyzed by *ATF1* encoding alcohol acetyltransferase. In our study, deletion of *OYE2* or *ATF1* led to a 1.8-fold increase of geraniol production. Interestingly, the *ATF1* deletion strain increased citronellol production compared with the control strain (Additional file 1: Figure S1). A similar phenomenon was observed in a previous study, which indicated that there may be a relationship between the physiological functions of *OYE2* and *ATF1*. Although the old yellow enzymes in yeast may have a physiological role in detoxification of unsaturated metabolites and reactive oxygen species, both *OYE2* and *ATF1* are thought to be involved in sterol metabolism [38, 39]. Therefore, functional complementation in the regulation of sterol metabolism between *OYE2* and *ATF1* may be a reason for the lower cell growth and geraniol production in the double deletion strain.

In the geraniol-producing engineered *S. cerevisiae*, the reaction catalyzed by *ERG20* is a key branch point towards either geraniol synthesis or downstream ergosterol synthesis for cell growth. Although *ERG20* mutations could improve monoterpene production, the reduction of intercellular FPP synthesis damaged cell growth [8, 16]. In this study, the weak promoter P_*BTS1*_ and the copper-repressible promoter P_*CTR3*_ were first tested for *ERG20* down-regulation to redirect GPP flux. The transcriptional level of *ERG20* in the promoter replacement strains was decreased to a one-fifth to one-sixth, and reduced cell growth was also observed when geraniol synthase and the MVA pathway were overexpressed in promoter replacement strains (Fig. 4; Additional file 1: Figure S4). The enhancement of the MVA pathway together with *ERG20* down-regulation possibly led to the accumulation of toxic intermediates such as DMAPP or GPP. The toxicity of isoprenoid precursors has also been reported in *E. coli* and *Bacillus subtilis* [40–42]. Previously, we found that although overexpression of *ERG20* did not affect growth, it decreased geraniol production. Therefore, neither up-regulation or down-regulation of *ERG20* was able to increase geraniol production. A suitable *ERG20* expression strategy that can weaken the downstream FPP flux but not impair cell growth to further improve geraniol production in engineered yeast is needed. To achieve this goal, dynamic control of *ERG20* is proposed, which is likely to not only balance metabolism between product formation and cell growth, but also prevent the accumulation of toxic metabolites. Recently, several dynamic control systems have been developed and applied in metabolic engineering, i.e., a biosensor (sensor-regulator) system [43], a sequential control system based on the medication *GAL* regulation system combined with the *HXT1* promoter [23] and a novel CRISPR-Cas9 interference system [44]. To avoid early accumulation of toxic intermediates and to better balance the utilization of GPP, P_*HXT1*_, a glucose-sensing promoter, was employed to dynamically control *ERG20* expression in our study. An *HXT1* promoter replacement strain (YZG19) did not contribute to improve geraniol production in batch or fed-batch fermentation using glucose as sole carbon, but its advantage appeared when the glucose concentration was low (feeding glucose/ethanol mixture carbon). The geraniol production improved stepwise when the glucose percentage decreased in the glucose/ethanol mixture (Figs. 4, 5). These results indicate that the dynamic control of *ERG20* expression by the *HXT1* promoter can balance the flux distribution between cell growth and monoterpene synthesis, thus improving geraniol production with low glucose feeding (Fig. 5). In addition, ethanol feeding possibly improved the geraniol production to some extent due to supplying acetyl-CoA precursors in a more direct metabolic pathway. An ethanol feeding strategy has also been successfully applied in amorphadiene and resveratrol production [34, 35].

Finally, strategies combining the *OYE2* deletion and P_*HXT1*_-controlled *ERG20* expression further improved geraniol production to 984.6 mg/L (Fig. 6). A previous report showed that leucine metabolism may be networked with sterol biosynthesis, and that leucine was disassimilated to form HMG-CoA in *Leishmania mexicana* [45]. In *S. cerevisiae*, the leucine biosynthetic pathway may act as a bypass pathway to supplement additional HMG-CoA. Therefore, we further constructed the prototrophic strain YZG21 by complementing the auxotrophic marker *LEU2*. The *LEU2* complementation ultimately improved the geraniol production to 1.69 g/L, meanwhile also improving cell growth and yield. A similar result was observed in a previous study on miltiradiene production [36].

Although a relatively high level of geraniol was obtained in our work, the cytotoxicity of monoterpenes is still considered as a problem that hinders further improvement of monoterpene production. Improving the resistance of microorganisms to monoterpenes still needs to be addressed to further enhance the monoterpene production. In addition, diploidization is another potential strategy for improving biomass and productivity given that diploid strains generally grow faster and tolerate higher stresses compared with haploid strains [36, 46]. More importantly, constructing a plasmid-free geraniol overproducing strain by integration of all pathway components into the genome is necessary for application in industrial processes.

## Conclusions

In summary, we engineered geraniol biosynthesis through the reduction of endogenous geraniol conversion and dynamic control of *ERG20* expression. In addition, we further identified the importance of *LEU2* complementation to geraniol synthesis. Ultimately, 1.69 g/L of geraniol was achieved when these approaches were combined in fed-batch fermentation with pure ethanol feeding. The present work provides a good platform for the production of geraniol and its derived chemicals.

## Methods

### Medium

The *E. coli* strain Tans5α was used for gene cloning and grown in Luria–Bertani medium (5 g/L yeast extract, 10 g/L tryptone, and 10 g/L NaCl) supplemented with 100 mg/L ampicillin at 37 °C. The engineered yeast strain YZG13-GE1 constructed in our previous work was used as the parent strain for further engineering [21]. Yeast cells were cultivated in yeast extract-peptone-dextrose (YPD) medium (20 g/L glucose, 20 g/L tryptone, and 10 g/L yeast extract), SD-URA or SD-URA-HIS medium (20 g/L glucose, 1.7 g/L yeast nitrogen base, and 5 g/L (NH_4_)_2_SO_4_; synthetic complete drop-out medium without uracil and/or histidine). Geneticin (G418) at 400 mg/L was added to the culture medium for gene deletion and promoter replacement.

### DNA manipulation and strain construction

All yeast strains constructed in this study are listed in Table 2. The reference strain is YZG13-GE1, which is derived from CEN.PK102-5B and harbored pZGV6-GE1 (2µ ori, *URA3*, P_*TEF1*_-*tVoGES*-(GGGS)-*ERG20* ^*WW*^) and pZMVA4 (2µ ori, *HIS3*, P_*TEF1*_-*tHMG1*, P_*PGK1*_-*IDI1*, P_*TEF1*_-*UPC2.1*) [21]. The primers for all DNA fragment amplifications are listed in Additional file 1: Table S1. To replace the *ERG20* promoter with different promoters, integration cassettes were constructed via fusion PCR using loxp-*kanMX*-loxp as the selection marker. The *BTS1* promoter (from −1 to −333), *CTR3* promoter (from +30 to −1116), and *HXT1* promoter (from −1 to 1190) replaced the *ERG20* promoter in the chromosome [23, 47, 48]. Similarly, the deletions of genes *ATF1* and *OYE2* were also performed through homologous recombination using loxp-*kanMX*-loxp as the selection marker. The *LEU2* cassette was integrated into the YPRCτ3 site of the chromosome. The DNA fragments were transformed into *S. cerevisiae* CEN.PK102-5B by the standard lithium acetate method [49], and the transformants were selected on YPD agar plates supplemented with 400 mg/L geneticin (G418). The plasmid pSH47 (purchased from Euroscarf) was transformed into *S. cerevisiae* to remove the selection marker. The engineered strains were then transformed with plasmids pZGV6-GE1 and pZMVA4, resulting in a series of geraniol-producing strains (Table 2).

**Table 2:** Strains used in this study

Strain name	Parent strain	Plasmids/genotype	Source
CEN.PK102-5B		*MATa ura3*-*52 his3Δ1 leu2*-*3,112*	Dr. P. Kötter, Frankfurt, Germany
5B-Δoye2	CEN.PK102-5B	Δ*oye2::loxP*	This study
5B-Δatf1	CEN.PK102-5B	Δ*atf1::loxP*	This study
5B-Δoye2Δatf1	CEN.PK102-5B	Δ*oye2::loxP,* Δ*atf1::loxP*	This study
5B-PBTS1	CEN.PK102-5B	Δ*P*_*ERG20*_ *::loxP*-*P*_*BTS1*_	This study
5B-PCTR3	CEN.PK102-5B	Δ*P*_*ERG20*_ *::loxP*-*P*_*CTR3*_	This study
5B-PHXT1	CEN.PK102-5B	Δ*P*_*ERG20*_ *::loxP*-*P*_*HXT1*_	This study
5B-Δoye2-PHXT1	5B-PHXT1	Δ*P*_*ERG20*_ *::loxP*-*P*_*HXT1*_, Δ*oye2::loxP*	This study
5B-LEU2-Δoye2-PHXT1	5B-Δoye2-PHXT1	Δ*P*_*ERG20*_ *::loxP*-*P*_*HXT1*_, Δ*YPRCtau3::P*_*LEU2*_-*LEU2*-*T*_*LEU2*_-*loxP*	This study
YZG13-GE1	CEN.PK102-5B	pZGV6-GE1, pZMVA4	[21]
YZG14	5B-Δoye2	pZGV6-GE1, pZMVA4	This study
YZG15	5B-Δatf1	pZGV6-GE1, pZMVA4	This study
YZG16	5B-Δoye2Δatf1	pZGV6-GE1, pZMVA4	This study
YZG17	5B-PBTS1	pZGV6-GE1, pZMVA4	This study
YZG18	5B-PCTR3	pZGV6-GE1, pZMVA4	This study
YZG19	5B-PHXT1	pZGV6-GE1, pZMVA4	This study
YZG20	5B-Δoye2-PHXT1	pZGV6-GE1, pZMVA4	This study
YZG21	5B-LEU2-Δoye2-PHXT1	pZGV6-GE1, pZMVA4	This study

### Batch and fed-batch fermentation for geraniol production

To determine the performance of the recombinant yeast strains, batch fermentation and fed-batch fermentation were performed in a 1-L Infors-HT fermenter (Infors AG, Bottmingen, Switzerland). To prepare seed cultures, the strains were grown for 24 h in 5 mL of SD-URA-HIS medium, and then inoculated into fresh SD-URA-HIS medium at an OD_600_ of 0.2 and cultivated for 12 h. For batch fermentation, a seed culture was inoculated into a 1-L Infors-HT fermenter (Infors AG, Bottmingen, Switzerland) containing 0.6 L of SD-URA-HIS medium at an initial OD_600_ of 0.2. Batch cultures were conducted at 30 °C, with the agitation rate at 600 rpm and an airflow rate of 1 vvm. The pH was maintained at 5.0 by automatic addition of 2.5 M NaOH. Dissolved oxygen was maintained above 30% saturation throughout cultivation by setting the stirring speed rate. For fed-batch cultivation, strains were first grown in a 400-mL batch culture at 30 °C with shaking at 600 rpm, an airflow rate of 1 vvm and pH 5.0. After the glucose and ethanol produced in the batch phase were depleted, feeding solution containing 400 g/L glucose, 100 g/L (NH_4_)_2_SO_4_, 34 g/L yeast nitrogen base, 1.3 g/L CSM-Ura-His, and 2 g/L leucine was fed to the fermenter at a controlled specific feed rate of 0.1/h. Feeding of a glucose/ethanol mixture or pure ethanol was also used for strains in which the *ERG20* promoter was replaced with the *HXT1* promoter to control *ERG20* expression. In both batch and fed-batch cultures, 20% dodecane was added to the medium after cultivating for 12 h for geraniol extraction. Independent duplicate cultures were conducted for each strain.

### RNA extraction and quantitative real-time PCR

Total RNA was prepared from 40 mL exponentially growing cell cultures using the UNIQ-10 Trizol RNA extraction kit (Sangon Biological Engineering, Shanghai, China). The samples were digested using DNase I treatment (TakaRa, Dalian, China) to avoid DNA contamination. The treated total RNA was used for cDNA synthesis using the PrimeScript RT-PCR Kit (TakaRa, Dalian, China). qPCR was performed using the SYBR Green Master Mix Kit (Roche Molecular Biochemicals, Germany). The *ACT1* gene was chosen as the internal control gene. The relative transcription levels of genes were analyzed using the 2^−ΔΔ*CT*^ method.

### Analysis of metabolites by HPLC

At each 12-h interval, the cultures were sampled and centrifuged at 12,000 rpm for 5 min. The filtered samples were analyzed using an HPLC equipped with an Aminex HPX-87H ion-exchange column (Bio-Rad, Hercules, CA, USA) at 45 °C with a mobile phase of 5 mM H_2_SO_4_ at a flow rate of 0.6 mL/min. The peaks of metabolites including glucose, ethanol, acetic acid and glycerol were detected by refractive index (RI) and ultraviolet (UV) detectors.

### Characterization of geraniol and citronellol by GC–MS

To quantify titers of geraniol and citronellol in different cultures, 1 mL of the upper layer (dodecane/culture mixture) was sampled and concentrated at 13,000 rpm for 5 min to separate the dodecane phase, and then the dodecane layer was transferred into a GC vial and stored at −20 °C for analysis. The residual geraniol was extracted again by adding 10% (v/v) dodecane into the medium. Geraniol and citronellol were identified using a GC–MS system (Shimadzu Co., Kyoto, Japan) equipped with an HP-5 ms capillary column (30 m × 0.25 mm × 0.25 µm), an AOC-20i auto-injector, and a QP-2010 mass detector, and the operational conditions were as follows. One microliter of each dodecane sample was injected into the system with a split ratio of 10 and the carrier gas helium was set at a constant flow rate of 0.78 mL/min. The oven temperature was first maintained at 60 °C for 2 min, and then gradually increased to 150 °C at a rate of 10 °C/min, held for 10 min, and finally increased to 230 °C at a rate of 20 °C/min and held for 5 min. The mass spectrometer was set to SIM acquisition mode, scanning m/z ions within the range 40–500 for identification of geraniol and citronellol. The total run time was 30 min. Standard compounds of geraniol and citronellol (Sigma-Aldrich) were dissolved in dodecane and used to plot standard curves for quantification.

### Biomass determination

Optical density at 600 nm (OD_600_) was measured using a spectrophotometer (Eppendorf AG, 22331 Hamburg, Germany). Dry cell weight (DCW) was obtained from OD_600_ measurement after calibration as indicated before ($1\;{\text{g/L biomass}} = 0.246 \times \left( {OD_{600} } \right) - 0.0012$) [50].

## Additional file

**Additional file 1: Table S1.** Primers used in this study. **Figure S1.** The conversion of geraniol to citronellol in control strain and deletion strains. The data shown are representative of duplicate experiments, and the error bars represent the standard deviation. **Figure S2.** The effect of *OYE2* or/and *ATF1* deletion on cell growth in batch fermentation. All strains harbored pZGV6-GE1and pZMVA4 plasmids for geraniol production. The data shown are representative of duplicate experiments, and the error bars represent the standard deviation. **Figure S3.** Transcription level of *ERG20* controlled by different promoters in SD-URA-HIS medium with different concentrations glucose. The cells were collected when OD600 reached 0.6 to extract mRNA for *ERG20* mRNA and transcription determination. The data shown are representative of triplicate experiments, and the error bars represent the standard deviation. **Figure S4.** The effect of *ERG20* expression controlled by different promoters on cell growth in batch fermentation. All strains harbored pZGV6-GE1and pZMVA4 plasmids for geraniol production. The data shown are representative of duplicate experiments, and the error bars represent the standard deviation.
