# Supplementary material for: Dynamic control of ERG20 expression combined with minimized endogenous downstream metabolism contributes to the improvement of geraniol production in Saccharomyces cerevisiae
Source: Microb Cell Fact. 2017 Jan 31;16:17. doi: 10.1186/s12934-017-0641-9 (PMC5282783; doi:10.1186/s12934-017-0641-9)
Supplement: Supplementary file 1 — Additional file 1: Table S1. Primers used in this study. Figure S1. The conversion of geraniol to citronellol in control strain and deletion strains. The data shown are representative of duplicate experiments, and the error bars represent the standard deviation. Figure S2. The effect of OYE2 or/and ATF1 deletion on cell growth in batch fermentation. All strains harbored pZGV6-GE1and pZMVA4 plasmids for geraniol production. The data shown are representative of duplicate experiments, and the error bars represent the standard deviation. Figure S3. Transcription level of ERG20 controlled by different promoters in SD-URA-HIS medium with different concentrations glucose. The cells were collected when OD600 reached 0.6 to extract mRNA for ERG20 mRNA and transcription determination. The data shown are representative of triplicate experiments, and the error bars represent the standard deviation. Figure S4. The effect of ERG20 expression controlled by different promoters on cell growth in batch fermentation. All strains harbored pZGV6-GE1and pZMVA4 plasmids for geraniol production. The data shown are representative of duplicate experiments, and the error bars represent the standard deviation. [file 12934_2017_641_MOESM1_ESM.pdf]

Supplementary information

Dynamic control of *ERG20* expression combined with minimized endogenous downstream metabolism contributes to the improvement of geraniol production in *Saccharomyces cerevisiae*

Jianzhi Zhao[1], Chen Li[1], Yan Zhang[1], Yu Shen[1], Jin Hou[1]*, Xiaoming Bao[1,2]*

[1] State Key Laboratory of Microbial Technology, School of Life Science, Shandong University, Jinan 250100, China

[2] Shandong Provincial Key Laboratory of Microbial Engineering, School of Bioengineering, QiLu University of Technology, Jinan, 250353, China

*Corresponding authors: Xiaoming Bao and Jin Hou. Tel/Fax: +86 531 8836 5826;
E-mail: bxm@sdu.edu.cn and houjin@sdu.edu.cn.

Table S1. Primers used in this study.

Primer name	Sequences (5’→3’)	Amplified genes
P_{ERG20}-UP-F	GACAATCATTACCACAAGATGAACAC	*ERG20* upstream arm
P_{ERG20}-UP-R	GCGTACGAAGCTTCACCGCTTAGAATACCTC ACACTG	
P_{ERG20}-DN-F	ATGGCTTCAGAAAAAGAAATTAGG	*ERG20* downstream arm
P_{ERG20}-DN-R	GTCGACTTTGTCTTCAGGTGC	
kanMX-F	AGGTATTCTAAGCGGTGAAGCTTCGTACGCT GCAGG	*KanMX* expression cassette
kanMX-R	CGCATAGGCCACTAGTGGATCTGAT	
P_{BTS1}-F	GTGATATCAGATCCACTAGTGGCCTATGCGA CGATGTATAGCCGCCATCTC	*BTS1* promoter
P_{BTS1}-R	CTCTCTCCTAATTTCTTTTTCTGAAGCCATTG ATTTTCCAGACTCGTAAAC	
P_{CTR3}-F	GTGATATCAGATCCACTAGTGGCCTATGCGA AGATAATAGACAGTCATAGCATGA	*CTR3* promoter
P_{CTR3}-R	CTCTCTCCTAATTTCTTTTTCTGAAGCCATAG CAGTGCTGCTACTGCCTC	
P_{HXT1}-F	GTGATATCAGATCCACTAGTGGCCTATGCGT GCAGGTCTCATCTGGAATATAATTCC	*HXT1* promoter
P_{HXT1}-R	CTCTCTCCTAATTTCTTTTTCTGAAGCCATGA TTTTACGTATATCAACTAGTTGAC	
OYE2-UP-F	AAAACGGAGTAGAATCGGTAAG	*OYE2* upstream arm
OYE2-UP-R	CGTCTATATTTAGCTTAATATGATG	
OYE2-DN-F	TAGTGTTAACCGTACTTTGTAG	*OYE2* downstream arm
OYE2-DN-R	ATAGGATGATGAATGACAGCAT	
ATF1-UP-F	ACTTTTTGGACATTGAGCTAAG	*ATF1* upstream arm
ATF1-UP-R	GAGAGCTGATAAATTGATGGT	
ATF1-DN-F	ATCTCACATGATGCTTGACTG	*ATF1* downstream arm
ATF1-DN-R	CGACGATTCTGACCCTTTCTA	
OYE2-kanMX-F	TAAATCATCATATTAAGCTAAATATAGACGTG AAGCTTCGTACGCTGCAGG	*OYE2* deletion fragment
OYE2-kanMX-R	AAATGGTGCTACAAAGTACGGTTAACACTAC GCATAGGCCACTAGTGGATCT	
ATF1-kanMX-F	ATCACAAATACCATCAATTTATCAGCTCTCTG AAGCTTCGTACGCTGCAGG	*ATF1* deletion fragment
ATF1-kanMX-R	GAATAATATCAGTCAAGCATCATGTGAGATC GCATAGGCCACTAGTGGATCT	
LEU2-F	CAAAGGGGACGTTCTTCACCTCCTTGGAAT GTGTTCCACTATCCTGTACATGAACTGTGGG AATACTCAGGTATCGT	*LEU2* expression cassette
LEU2-R	CAAATATATTCCATGGCCTCTTAGTTTGGCAA CCCAAGACTCGGCATACCTCGACTACGTCGT TAAGGCCG	

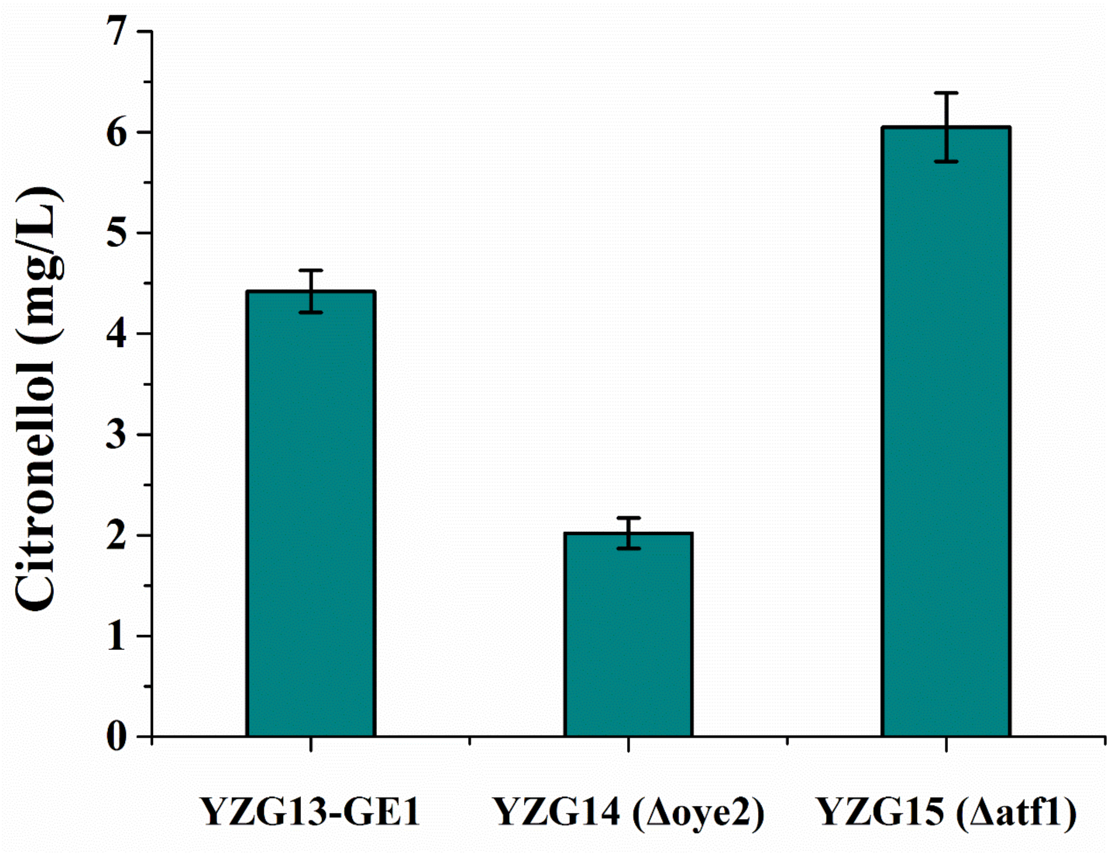

Figure S1. The conversion of geraniol to citronellol in control strain and deletion strains. The data shown are representative of duplicate experiments, and the error bars represent the standard deviation.

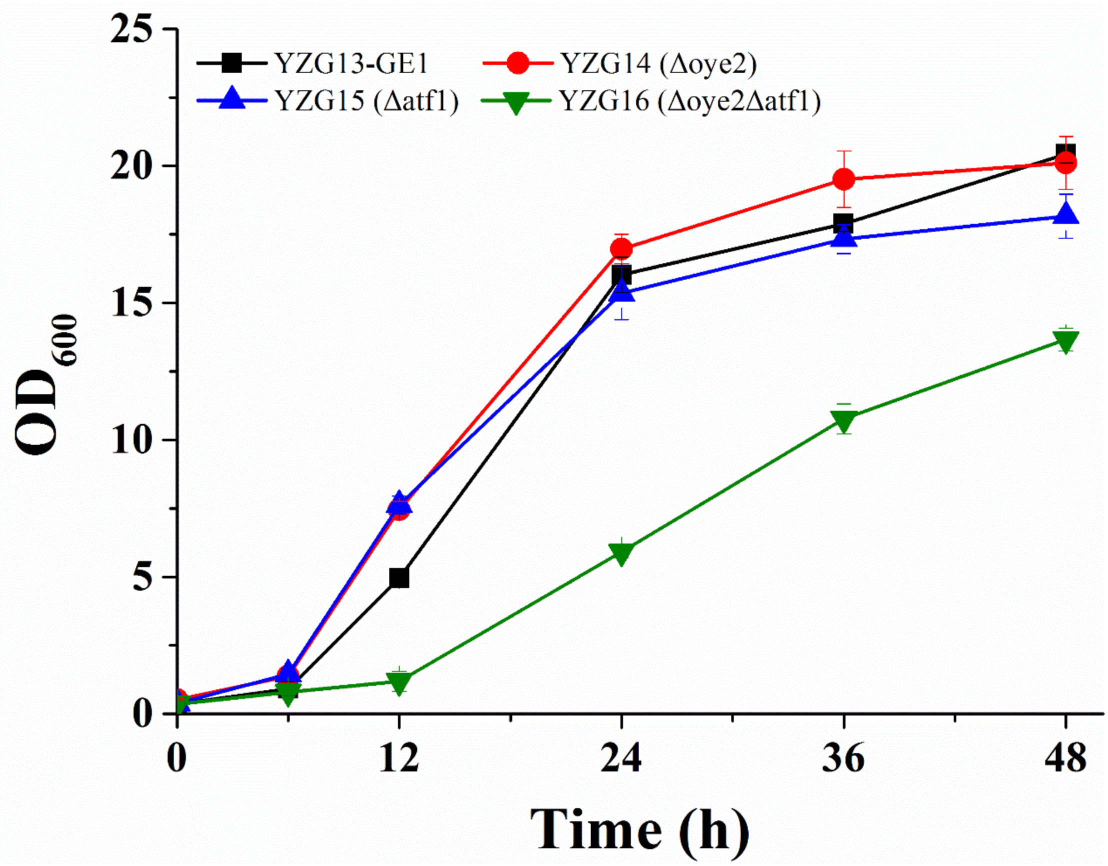

Figure S2. The effect of *OYE2* or/and *ATF1* deletion on cell growth in batch fermentation. All strains harbored pZGV6-GE1and pZMVA4 plasmids for geraniol production. The data shown are representative of duplicate experiments, and the error bars represent the standard deviation.

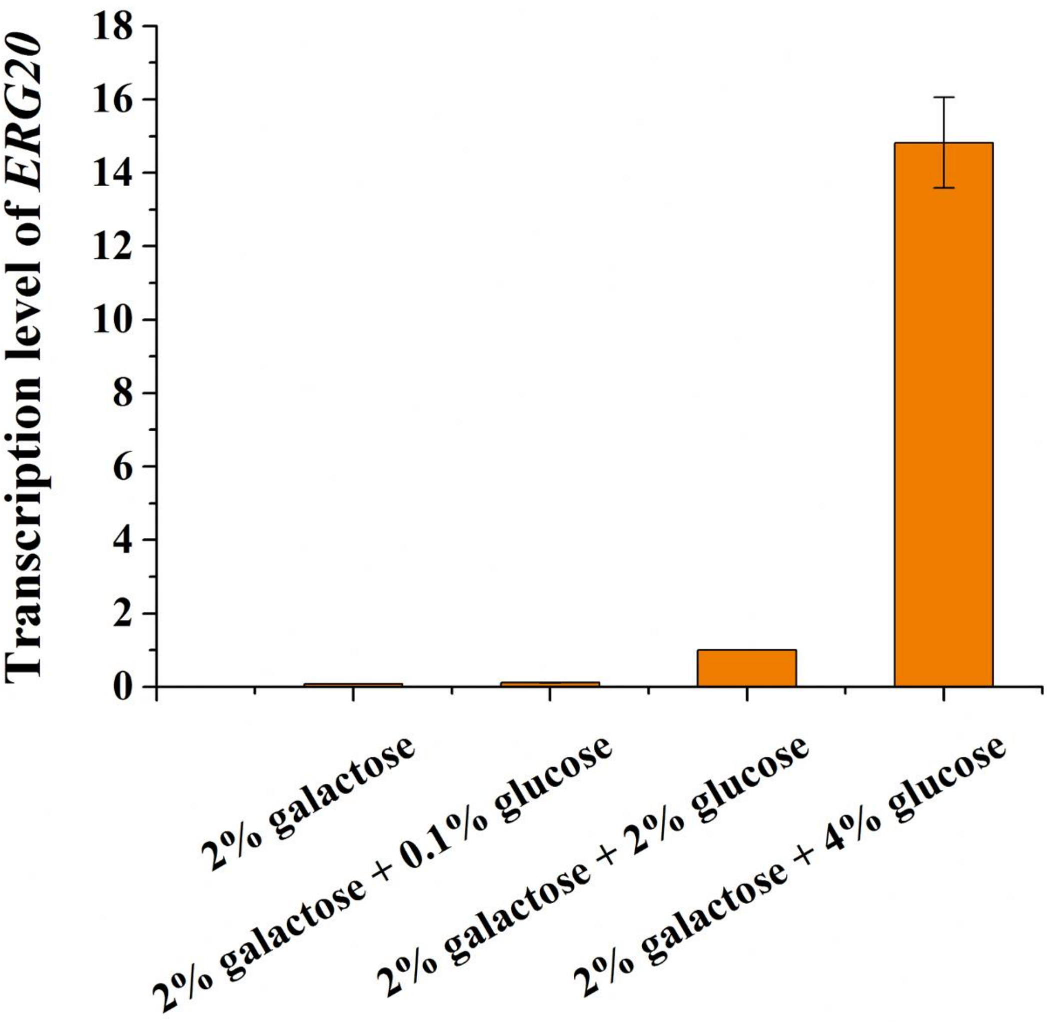

Figure S3. Transcription level of *ERG20* controlled by different promoters in SD-URA-HIS medium with different concentrations glucose. The cells were collected when OD_{600} reached 0.6 to extract mRNA for *ERG20* mRNA and transcription determination. The data shown are representative of triplicate experiments, and the error bars represent the standard deviation.

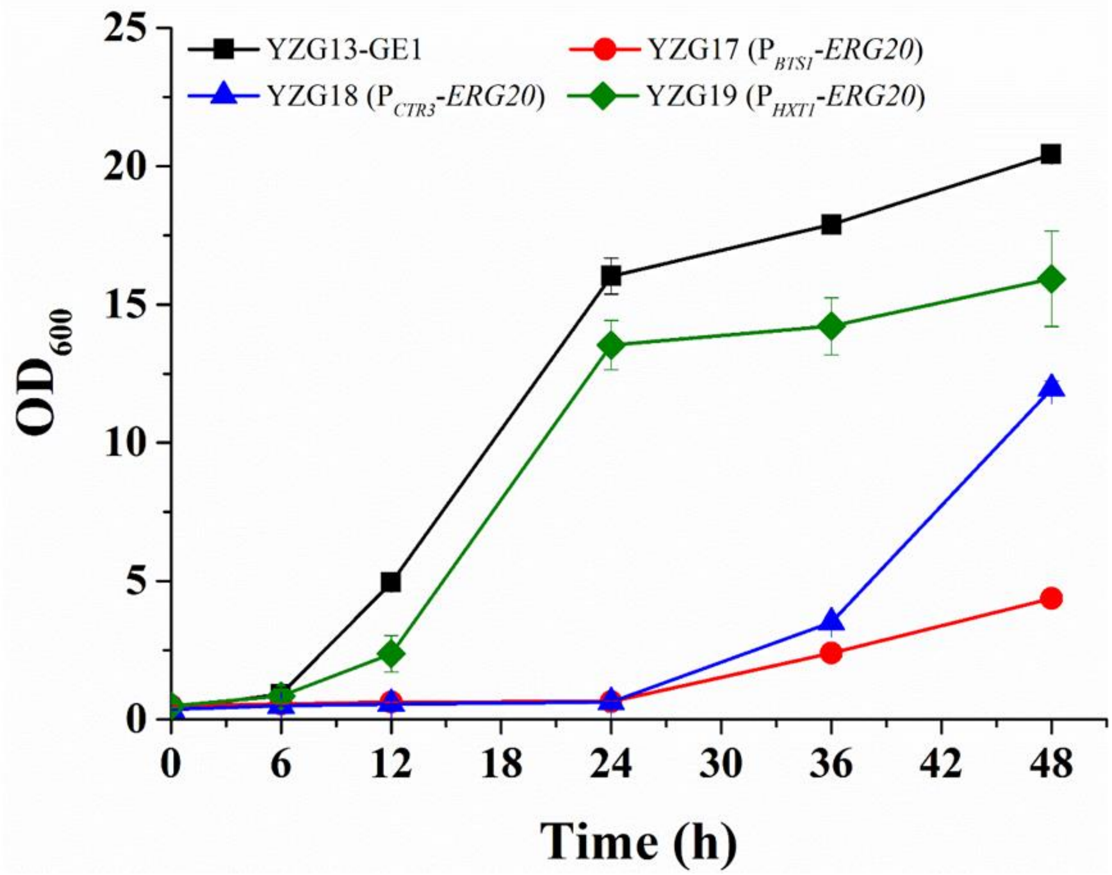

Figure S4. The effect of *ERG20* expression controlled by different promoters on cell growth in batch fermentation. All strains harbored pZGV6-GE1and pZMVA4 plasmids for geraniol production. The data shown are representative of duplicate experiments, and the error bars represent the standard deviation.
